# Supplementary material for: Phototaxis as a Collective Phenomenon in Cyanobacterial Colonies
Source: Sci Rep. 2017 Dec 19;7:17799. doi: 10.1038/s41598-017-18160-w (PMC5736714; doi:10.1038/s41598-017-18160-w)
Supplement: Supplementary file 1 — Supplementary Information [file 41598_2017_18160_MOESM1_ESM.pdf]

# SUPPLEMENTARY INFORMATION

## 'Phototaxis as a Collective Phenomenon in Cyanobacterial Colonies'

P. Varuni, Shakti N. Menon and Gautam I. Menon

### Supplementary Figure

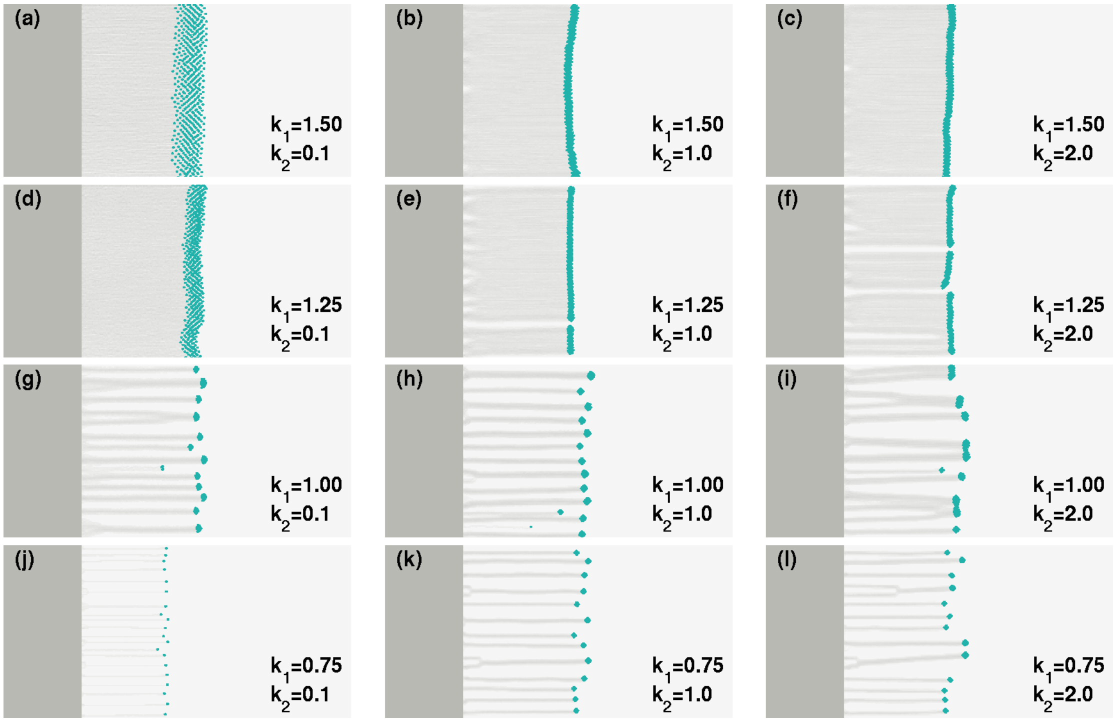


Figure S1: **Effect of changing the parameters $k_1$ and $k_2$:** Snapshots of the colonies at $t = 10^5$ for different values of the parameters $k_1$ and $k_2$ for the situation where a light source is placed at infinity (to the right of the colony). (a-f) For high $k_1$, the colony move as a front. (g-i) At $k_1 = 1$ we observe finger-like projections. (j-l) At even lower $k_1$, cells move individually towards the light source. The parameter $k_2$, however, only has a small effect on the morphology of the phototactic response, as observed by going from the left to right panels of each row.

## Supplementary Movies

Movie S1: Time lapse movie of a circular colony moving toward light showing the positions of individual cells and the slime they lay down over the course of a simulation. We start with a circular colony of cells of density $\rho = 0.015$. All other parameters are the same as that specified in Table 1 of the main text. Each subsequent frame is separated by 200 time steps.

Movie S2: Time lapse movie of a flat colony moving toward light showing the positions of individual cells and the slime they lay down over the course of a simulation. We start with a circular colony of cells of density $\rho = 0.05$. The cells encounter a slime band along their path. All other parameters are the same as that specified in Table 1 of the main text. Each subsequent frame is separated by 200 time steps.

Movie S3: Time lapse movie of a circular colony moving toward light showing the positions of individual cells and the slime they lay down over the course of a simulation. We start with a circular colony of cells of density $\rho = 0.015$. Once fingers are formed, the position of light (indicated by a yellow star) is changed. All other parameters are the same as that specified in Table 1 of the main text. Each subsequent frame is separated by 200 time steps.
