# Supplementary figures and images for: Phototaxis as a Collective Phenomenon in Cyanobacterial Colonies
Source: Sci Rep. 2017 Dec 19;7:17799. doi: 10.1038/s41598-017-18160-w (PMC5736714; doi:10.1038/s41598-017-18160-w)

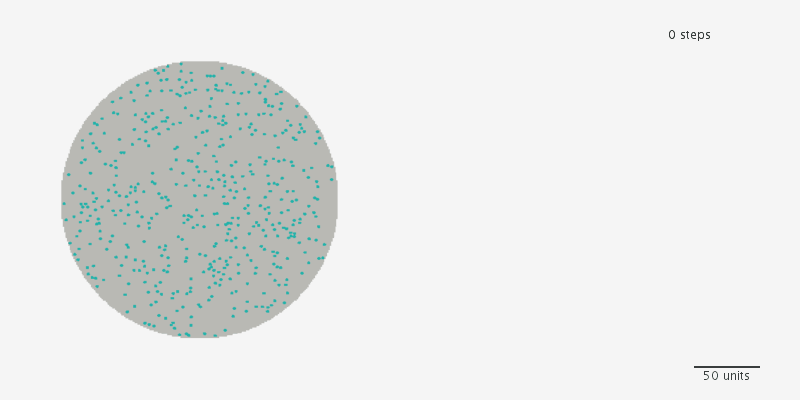

Supplement: Supplementary file 2 — Movie S1 [file 41598_2017_18160_MOESM2_ESM.gif]

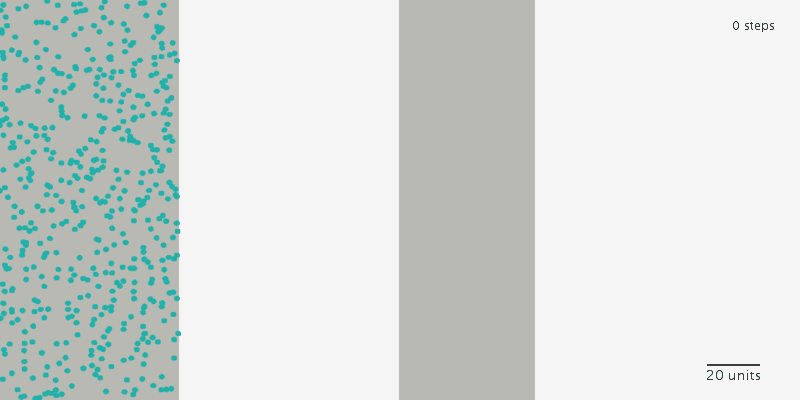

Supplement: Supplementary file 3 — Movie S2 [file 41598_2017_18160_MOESM3_ESM.gif]

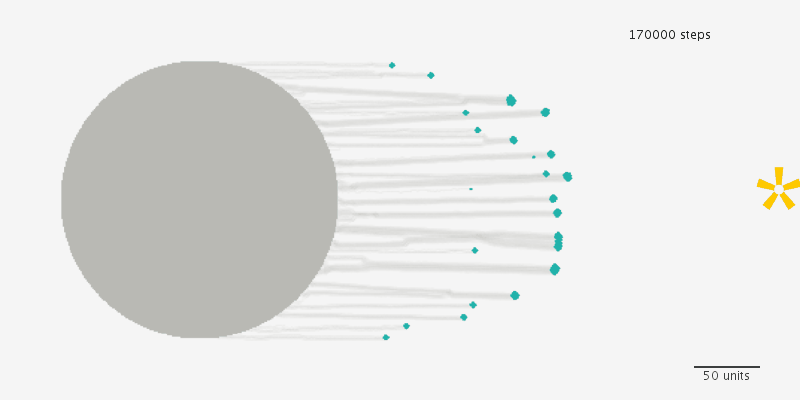

Supplement: Supplementary file 4 — Movie S3 [file 41598_2017_18160_MOESM4_ESM.gif]
